# Satellite DNA-like repeats are dispersed throughout the genome of the Pacific oyster *Crassostrea gigas* carried by *Helentron* non-autonomous mobile elements

**DOI:** 10.1038/s41598-020-71886-y

**Published:** 2020-09-15

**Authors:** Tanja Vojvoda Zeljko, Martina Pavlek, Nevenka Meštrović, Miroslav Plohl

**Affiliations:** Division of Molecular Biology, Ruđer Bošković Institute, Bijenička 54, 10 000 Zagreb, Croatia

**Keywords:** Mobile elements, Eukaryote, Evolution, Genetics

## Abstract

Satellite DNAs (satDNAs) are long arrays of tandem repeats typically located in heterochromatin and span the centromeres of eukaryotic chromosomes. Despite the wealth of knowledge about satDNAs, little is known about a fraction of short, satDNA-like arrays dispersed throughout the genome. Our survey of the Pacific oyster *Crassostrea gigas* sequenced genome revealed genome assembly replete with satDNA-like tandem repeats. We focused on the most abundant arrays, grouped according to sequence similarity into 13 clusters, and explored their flanking sequences. Structural analysis showed that arrays of all 13 clusters represent central repeats of 11 non-autonomous elements named *Cg_HINE,* which are classified into the *Helentron* superfamily of DNA transposons. Each of the described elements is formed by a unique combination of flanking sequences and satDNA-like central repeats, coming from one, exceptionally two clusters in a consecutive order. While some of the detected *Cg_HINE* elements are related according to sequence similarities in flanking and repetitive modules, others evidently arose in independent events. In addition, some of the *Cg_HINE*’s central repeats are related to the classical *C. gigas* satDNA, interconnecting mobile elements and satDNAs. Genome-wide distribution of *Cg_HINE* implies non-autonomous *Helentrons* as a dynamic system prone to efficiently propagate tandem repeats in the *C. gigas* genome.

## Introduction

Satellite DNAs (satDNAs) and transposable elements (TEs) are two types of repetitive sequences that together represent the largest fraction of eukaryotic genomes^1^. SatDNAs are head-to-tail tandemly repeated non-coding DNA sequences primarily organized in long arrays associated with heterochromatin. Nevertheless, satDNA sequences can also be found dispersed in the euchromatic genome fraction as short arrays, single monomers or their fragments (reviewed in^2–4^). While satDNAs appear relatively static with respect to their localization on chromosomes, TEs are sequences able to move throughout the genome, ultimately forming interspersed repeats. TEs spread by a variety of mechanisms, RNA-mediated (Class I elements), and DNA-mediated (Class II elements), either autonomously or dependent on enzymes produced by the autonomous elements^5,6^.

Regardless of the conceptual differences between satDNAs and TEs, numerous studies show that they can be interconnected in many different ways^7^. Thus, satDNA can be formed by tandem amplification of an entire TE or its part^8–11^. TEs themselves may have an internal region composed of sequences repeated in tandem. One example is *Tetris*, described in *Drosophila* as modularly structured non-autonomous foldback DNA transposon. It incorporates tandem repeats (TRs) that can act as building blocks in the formation of classical satDNA arrays^12^.

Among TEs that may contain TRs in their structure one group stands out, *Helitrons*, a diverse superfamily of DNA transposons widespread in animals and plants^13^. These elements use rolling-circle replication (RCR) to spread through the genome, and they do not create target site duplications (TSD) upon insertion^14^. Because of the RCR mechanism, they are prone to capture and propagate diverse genomic sequences, including genes, contributing significantly to genome evolution^15–17^. Whole elements can also be repeated in tandem, in which case the inner copies are often truncated at the 3′ end^18,19^. One structural variant of the *Helitron* superfamily is *Helentron*, in its non-autonomous form known as *HINE* (*Helentron*-associated INterspersed Elements). *HINEs* are characterized by two modules which include subterminal inverted repeats and a short palindromic sequence at the 3′ end of the right module. A short array of satDNA-like TRs can be often embedded between these two modules^20,21^.

Bivalve mollusks constitute a large class of marine and freshwater invertebrates carrying high ecological and commercial value^22^. The genome of the Pacific oyster *Crassostrea gigas* Thunberg, 1793 was the first sequenced and assembled bivalve genome. Because of high individual polymorphism and abundant repetitive sequences, estimated to build 36% of the genome, fosmid pooling, next-generation sequencing, and hierarchical assembly were combined in this work^23^. *C. gigas* genome is replete with transposase and reverse-transcriptase gene fragments and their transcripts, indicating importance of transposition processes in shaping the genome^23,24^. In a recent analysis of the black-shelled Pacific oyster (a variety of the Pacific oyster *C. gigas*), long and short reads sequencing was combined and even a higher content (48%) of repetitive sequences was revealed than in the previous study^25^. It was concluded that both strains are highly variable and divergent in content of their repetitive sequences.

*C. gigas* has a low level of heterochromatin observed as a weak centromeric C-band on one chromosome pair, and a telomeric C-band on the other^26^. This observation is in agreement with the low abundance of classical satDNAs, as predicted by Zhang et al.^23^. The most abundant satDNA in *C. gigas* is Cg170, with ~ 166 bp long monomers, occupying 1–4% of the genome, and detected in centromeric regions of only some chromosome pairs^27,28^. A similar satDNA was identified independently in seven oyster species belonging to the genera *Ostrea* and *Crassostrea*, and named HindIII satDNA denoting the restriction site by which it was detected^29^. Although described independently, both satDNAs can be considered as subfamilies of one divergent family that can be unified under the name Cg170/HindIII^30^. A preliminary study on a limited sample of Cg170 satDNA arrays extracted from the genome assembly indicated their association with members of the *Helitron superfamily*^31^. Furthermore, Cg170/HindIII monomers are similar to the central repeats of the miniature inverted-repeat element (MITE) *Pearl*, which is widespread in bivalves^32^, and according to its structural characteristics was later re-categorized as a non-autonomous *Helentron*^21^.

A detailed view of the genomic inventory of satDNAs started to accumulate in different animal and plant species by combining advanced sequencing methods and specialized bioinformatics tools (for example^3,33–37^). However, information about content, distribution, and composition of short arrays of TRs located in euchromatic genome compartments and related or resembling satDNAs (therefore named satDNA-like sequences), and about genome environment in which they reside, remains limited and shown on a few species, mostly *Drosophila* and beetles^34,38–42^.

In this work, the genome assembly of *C. gigas*^23^ was searched for all TRs that resemble satDNAs according to criteria of monomer length. This strategy revealed short arrays of TRs dispersed throughout the genome. We focused on the 13 similarity-grouped clusters of most abundant arrays (49% of all detected), studied their adjacent genomic sequences, and found that they altogether can be characterized as non-autonomous elements of the *Helentron* superfamily (*HINE*). According to our best knowledge, by starting from a general inventory of satDNA-like TRs in a putative euchromatic (assembled) genome fraction, we show for the first time that divergent satDNA-like arrays of one species are all linked with *HINE* TEs as their carriers. In total, we identified a family of 11 elements, determined by flanking sequences associated with arrays assigned only to one or, exceptionally, two clusters of satDNA-like TRs.

## Results

### Detection and grouping of tandem repeats in *C. gigas*

**Figure 1:**
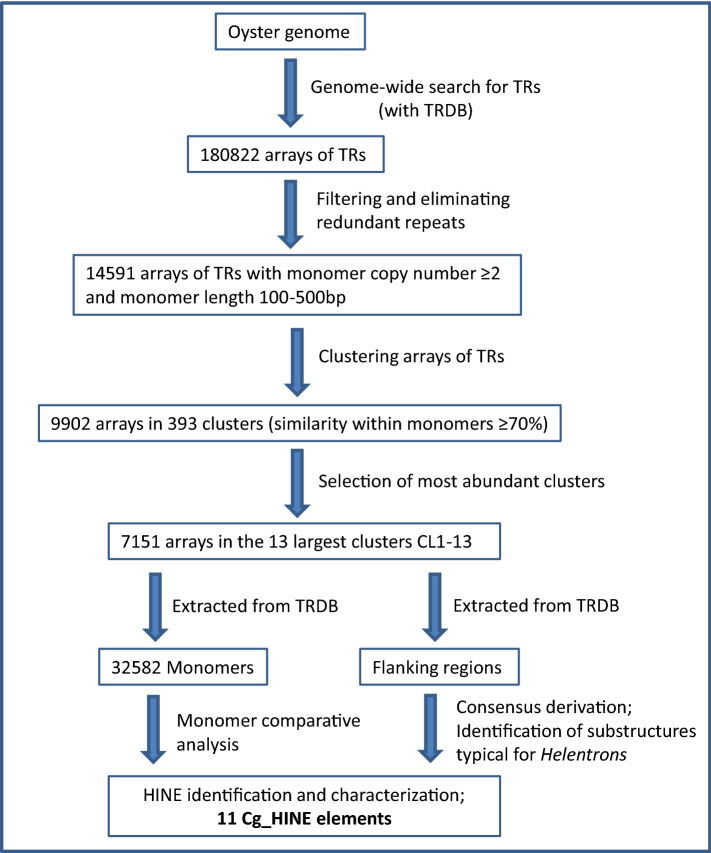
Workflow of the genome-wide identification of tandem repeats and their flanking sequences in the assembled genome of the Pacific oyster *Crassostrea gigas*.

The strategy used to detect and characterize satDNA-like TRs and their flanking sequences in the *C. gigas* genome is shown in Fig. 1.

**Figure 2:**
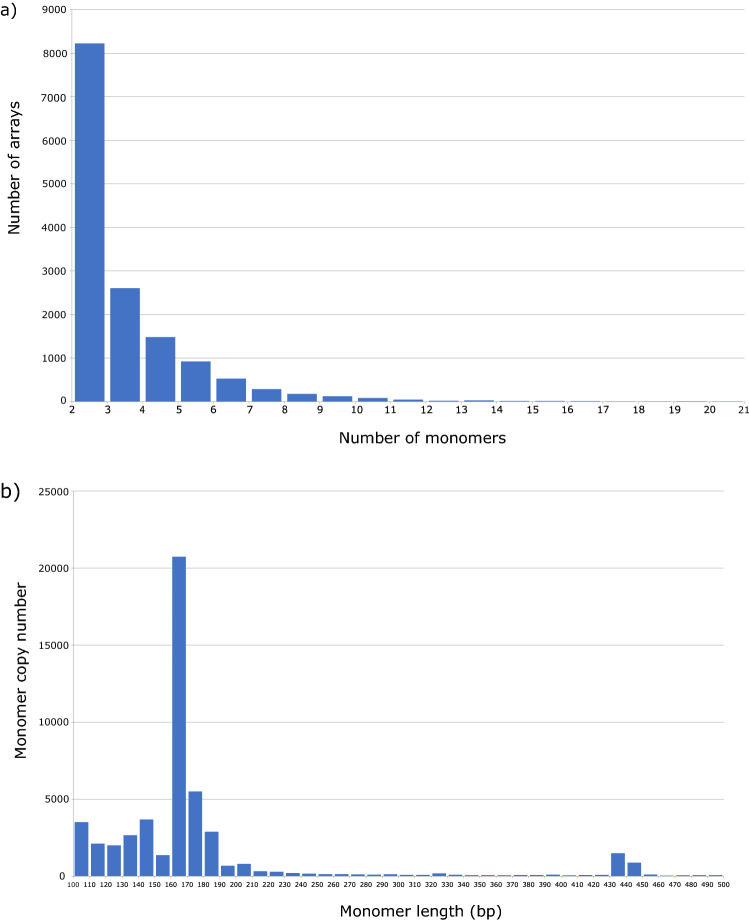
Correlation between number of monomers, monomer length, and number of arrays. Number of arrays plotted as a function of number of monomers (**a**), monomer copy number plotted as a function of monomer length (**b**).

Screening of the sequenced genome (oyster.v9.fa) and filtering out TRs composed of at least two monomers of the length between 100 and 500 bp revealed 14,591 arrays. Comparison of the number of arrays with the number of monomers in the array shows that the most abundant are those with 2 or 3 monomers (8,224 out of 14,591, or 56.36%; Fig. 2a). The number of detected arrays drops dramatically with increasing number of monomers, and only 2,282 (15.64%) arrays hold ≥ 5 monomers. In total, the set of 14,591 arrays is composed of 51,024 monomers; among them, those with length between 160 and 180 bp are dominant, constituting 51.41% of all monomers (Fig. 2b).

The selected arrays were grouped using 70% sequence similarity as the threshold value (Fig. 1). Grouping resulted in 393 clusters composed of 9,902 arrays, while the remaining 4,689 arrays were too divergent to allow clustering under these conditions. For further analysis we selected the 13 largest clusters, CL1-13, encompassing 7,151 arrays and representing the majority (72%) of clustered arrays (Supplementary Table S1). Altogether, arrays of TRs in clusters CL1-13 constitute ~ 4.8 Mb or 0.85% of the sequenced *C. gigas* genome (Table 1).

**Table 1:** Features of arrays of TRs grouped by similarity in 13 clusters in the genome of the Pacific oyster *Crassostrea gigas*.

Cluster	Number of arrays	Arrays with ≥ 5 monomers (% of total arrays)	Total length of arrays (kb)	Proportion in the genome assembly (%)**	Total number of monomers	Average number of monomers / array	Average GC (%)	Consensus monomer length (bp)
CL1	1,397 (900*)	507 (36.29%)	1,067,806	0.19	6,432 (4,881*)	4.6	39.22	167
CL2	1,031 (950*)	453 (43.94%)	855,355	0.15	5,183 (5,985*)	5.05	36.85	166
CL3	703	234 (33%)	531,465	0.1	3,209	4.56	31.16	167
CL4	684	167 (24.4%)	460,902	0.08	2,754	4.03	36.51	170
CL5	643	62 (9.64%)	359,046	0.06	2005	3.12	31.6	181
CL6	598	123 (20.5%)	346,142	0.06	2,384	3.99	39.57	147
CL7	564	42 (7.44%)	345,312	0.06	1957	3.47	41.24	178
CL8	488	47 (9.63%)	263,021	0.05	1635	3.35	32.96	162
CL9	307	25 (8.14%)	182,211	0.03	1,059	3.45	33.2	173
CL10	227	64 (28.19%)	153,409	0.03	927	4.08	34.66	167
CL11	213	0	82,801	0.01	509	2.39	30.91	162
CL12	156	1 (0.64%)	47,771	0.01	356	2.28	44.92	138
CL13	140	4 (2.86%)	62,802	0.01	380	2.72	32.29	168
Total CL1-13	7,151	1729	4,758,043	0.85	32,582	3.62	35.77	165

*Due to technical constrains of the Tandem Repeat Database, it was not possible to extract CL1 and CL2 monomers which would belong to all detected arrays, but from the majority of arrays. For the monomer consensus derivation, the maximal number of monomers which could be obtained for those two clusters was used: 4,881 CL1 monomers (belonging to 900 arrays of CL1) and 5,985 CL2 monomers (belonging to 950 arrays of CL2). **The calculation for the proportion of analyzed arrays (bp) in the genome assembly was done using the genome assembly size of 559 Mb^23^.

**Figure 3:**
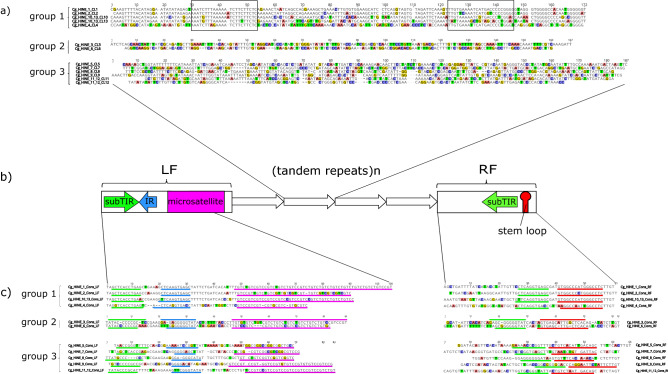
Structural characteristics and sequence comparisons of *Cg_HINE* elements. Consensus sequences of monomers belonging to each cluster are shown in (**a**). A general scheme of all depicted *CG_HINE* elements is presented in the central part of this figure (**b**). Consensus sequences of left and right flanking sequences (LF and RF, respectively) are presented in (**c**). Furthermore, elements are grouped according to sequence similarity. Group 1 form *Cg_HINE*s that share similarity in all element parts. In group 2 there are elements similar in flanking segments but not in monomers building TRs. Group 3 form elements divergent in their nucleotide sequences. Sequence segments corresponding to the structural elements in LF and RF are underlined with the same color as used in schematic presentation in (**b**). In group 1 monomer consensus sequences (**a**) boxed are sequence segments with reduced variability compared to monomers from the cluster CL4 (see text for explanation). In all alignments, differences present in less than half of nucleotides at each position are colored.

From clusters CL1-13 we extracted a total of 32,582 monomers. Monomer consensus sequences were derived by multiple sequence alignment of all repeats in each cluster (with exceptions for the most abundant CL1 and CL2, where > 70% repeats were used; Table 1). Alignment of the monomer consensus sequences is presented in Fig. 3a.

The monomer length in the studied clusters is predominantly 140–180 bp (Table 1), in agreement with the preferred length detected in the analysis of the initial set of 51,024 monomers. Exceptions are the monomers in clusters CL6 and CL12, with an average length of 146 and 138 bp, respectively. The average monomer copy number in arrays of clusters CL1-13 is up to 5, slightly higher than that shown for the whole set. In particular, arrays with ≥ 5 monomers are more abundant in clusters CL1, CL2, CL3, CL4, CL6, and CL10 (Table 1). The longest array in the whole set, with 29.3 monomers, was found in the cluster CL2.

### Comparisons of tandem repeats from clusters CL1-13

Nucleotide sequence diversity among monomers within a cluster ranges from 8.5% in CL9 to 21% in CL4, and differences among variants are mostly due to nucleotide substitutions. Sequence comparisons revealed that only monomers from clusters CL1, CL2, CL10, and CL13 share relevant similarity along the whole length (Group 1 in Fig. 3a). Particularly, CL1 and CL2 monomers are 89.8% identical in their consensus sequences, while they share ~ 70% identity with those from clusters CL10 and CL13 (Supplementary Table S3). Phylogenetic analysis confirmed, even in the case of the highly similar CL1 and CL2, grouping of monomers into four clearly distinctive clusters and homogeneity of arrays (Supplementary Fig. S1). Similarity is also shared between consensus sequences of monomers from clusters CL1, CL2, CL10, and CL13 and consensus sequences of Cg170^27^ and HindIII satDNAs^29^ (Supplementary Table S3). In this regard, monomers from these clusters can be considered as novel subfamilies of the Cg170/HindIII satDNA family. In addition, CL1, CL2, CL10, and CL13 share two relatively conserved sequence segments with monomers from the cluster CL4 (boxed segments in Group 1, Fig. 3a), although the rest of their sequence is dissimilar (see also Supplementary Table S3).

Our survey also revealed that the CL4 monomer consensus sequence is 97.6% similar to that of SAT-2_CGi, the *C. gigas* DNA sequence annotated in Repbase as a satDNA. However, according to our knowledge, this satDNA was not further characterized. A more detailed insight into the SAT-2_CGi Repbase entry (668 bp) revealed 4 tandemly repeated monomers, about 168 bp long, sharing 95.4% of mutual similarity^43^. Monomer consensus sequences from other clusters did not reveal similarities with any known satDNA.

### Tandem repeats from clusters CL1-13 are parts of *Cg_HINE* mobile elements

To characterize the genomic environment in which the detected satDNA-like arrays reside, we analyzed their flanking regions. Alignments of sequences flanking satDNA-like arrays revealed 50 to 100 bp long stretches of similarities that enabled derivation of left and right consensus sequences (LF and RF, respectively) for each cluster (Fig. 3b, c). These sequences were used as queries in a search through Repbase (Supplementary Table S4), which indicated high similarities with non-autonomous mobile elements assigned as members of the *Helitron* superfamily from *C. gigas*^43^.

**Figure 4:**
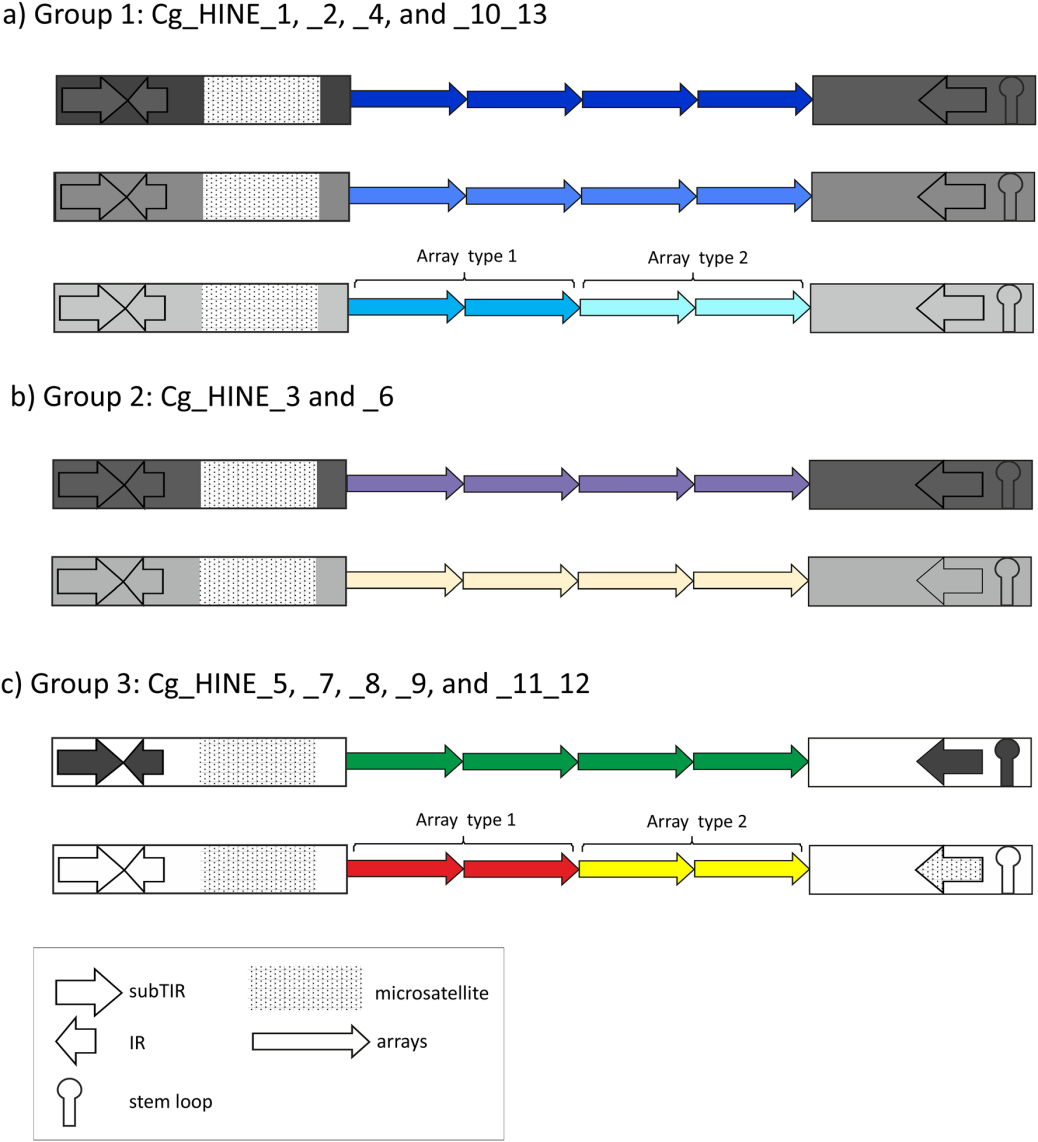
Schematic presentation of groups of *Cg_HINE* elements. Related elements of group 1 are shown in (**a**), related in flanking sequences but with divergent TRs of group 2 are in (**b**), and divergent in all segments (group 3) are in (**c**). Shown are also elements with two arrays of TRs, detected in group 1 and 3. Grey tones indicate sequence similarity in flanking segments. Tones of a color in monomers indicate similarity, while different colors indicate unrelated monomer sequences.

To explore the features of sequences that flank satDNA-like arrays in more detail, we excluded the array part, and, separately for each element, constructed LF-RF chimeric segments. The LF consensus sequence regularly ends with a microsatellite-like segment, separated from the first repeat in the array of TRs by a 10 to 500 bp long segment that is highly variable in DNA sequence and length. RF consensus sequence was identified as a 60 bp long segment following the 3′ end of the last repeat in an array, sharing > 80% similarity among elements within each cluster (Fig. 3c). The consensus sequences of these identified elements have typical HINE substructures^20^: 5′ subTIR, IR (complementary to the subTIR), and a microsatellite in LF; and 3′ subTIR and a palindrome in RF (Fig. 3b, Supplementary Table S2). The presence of these substructures was confirmed in the majority of array flanking regions (Supplementary Table S2). Accordingly, we named the identified *C. gigas* elements *Cg_HINE*. In total, we were able to distinguish 11 different *Cg_HINE* elements that carry arrays of TRs from clusters CL1-13. Exceptionally, sequences flanking arrays in clusters CL10 and CL13, and in clusters CL11 and CL12, were analyzed together owing to the fact that annotations revealed a predominant organization of these pairs of arrays in a consecutive order (Fig. 4).

Detailed analyses showed that perfect subTIRs are present in the majority of *Cg_HINE_9* and *Cg_HINE_10_13* elements, and 1 mismatch on different positions dominates in the rest. In all other elements subTIRs with 1 mismatch are predominant, while perfect subTIR sequences were found in < 50% of the examined flanking sequence pairs. The subTIR sequence, normally 11–12 bp long, is in LFs accompanied by 7–12 bp long complementary IR sequence, separated by a short segment of 1–12 bp. In turn, the subTIR in RF is followed by a palindrome (Fig. 3b, c, Supplementary Table S2). All of the examined 3′ palindromes have the potential of forming stem and loop structures (not shown).

Microsatellite sequences are mostly built of 4-nucleotide motifs as repeat units, although those with 3- and up to 8-nucleotide repeats were found (Supplementary Table S2). The LF sequence of several elements (*Cg_HINE_1, _3, _6, _8, _10_13, and _11_12*) share similar microsatellite sequences (GTCY, GTCC and GTCK). In all examined elements, the microsatellite array is short and highly variable in length and nucleotide sequence. Many ambiguous nucleotide positions, insertions and deletions in the nucleotide sequence indicate high level of mutations eroding this segment (Fig. 3c).

### Grouping of detected *Cg_HINE* elements

We further grouped *Cg_HINE* elements according to sequence similarities among their flanking segments and/or among their constitutive TRs, taking also into consideration the organizational patterns of TRs within elements (Fig. 4).

The group 1 (*Cg_HINE_1, _2, _4, and _10_13* in Fig. 3), characterized by similarity that extends throughout all element modules, is presented schematically in Fig. 4a. Related subfamilies of Cg170 satDNA are comprised of central repeats of elements in this group (Fig. 3a, Supplementary Table S3). Differences among TRs are accompanied by element-specific diagnostic changes in LF and RF consensus sequences, including in the subTIR, IR, stem and loops, and microsatellites (Fig. 3c, Supplementary Table S2). The highest similarity is between *Cg_HINE_1 and _2* which share the most similar monomers in their TRs (Supplementary Table S3). The most divergent in this group is *Cg_HINE_4*, sharing with others sequence similarity in the substructures of flanking segments (Fig. 3c), while its monomers are divergent, except in the two motifs shared with the rest (Fig. 3a). This group also includes *Cg_HINE_10_13*, which carries two consecutive arrays of related monomers, CL10 and CL13, instead of only one (Fig. 4a, Supplementary Table S3). The two arrays continue directly one after the other in the majority of *Cg_HINE_10_13* elements, although in a small fraction (~ 5%) they are separated by up to about 1.2 kb of anonymous DNA sequences.

Group 2 includes two elements, *Cg_HINE_3* and *_6*. In their flanking regions they differ in a way comparable to that of elements of the first group, although their TRs are completely unrelated (Fig. 3, 4b).

Elements *Cg_HINE_5, _7, _8*, and *_9* make up group 3 (Fig. 3, 4c). Modules of elements in this group are unrelated among themselves and with any other element in the studied sample. In each of these elements, satDNA-like TRs are derived from a single cluster of unique sequences, except in *Cg_HINE_11_12* which incorporates two arrays of TRs in a manner as described for *Cg_HINE_10_13* but with unrelated monomers (Fig. 4c).

We have also addressed the question of orientation of TRs with regard to their flanking sequences in the *Cg_HINE* element. Alignment of all elements shows the same orientation of flanking regions and their corresponding TRs, thus emphasizing the regularity of the proposed organizational pattern. This feature was observed in all studied elements without exception.

### Insertion sites and genomic distribution of *Cg_HINE* elements

To characterize insertion sites of *Cg_HINE*s, we constructed “empty site” chimeric fragments (i.e. without the *HINE* element) coupled at the element ends determined previously. For this, 50 bp long stretches upstream and downstream of an element were taken and used in a BLAST search throughout the genome assembly. Combining this analysis and inspecting the LF and RF consensus sequences, an insertion preference for T-rich regions was observed for all *Cg_HINE* elements. In general, TT dinucleotides are suggested to be the preferential insertion site (Supplementary Fig. S2). Furthermore, we did not find any indication of TSD at the insertion site. These two features are consistent with both *Helentrons* and *Helitrons*^21,44^.

To find *C. gigas* genomic sequences uninterrupted with *Cg_HINE* elements, we used “*Cg_HINE*-empty” chimeric constructs as a query. This search revealed the existence of uninterrupted segments of high similarity (> 90%) in the genome. For some chimeric constructs, the results disclosed many highly similar or identical hits, indicating insertions of *Cg_HINE*s into repetitive regions. Some of them could be identified as fragments of other TEs (mostly DNA transposons, but also some non-LTR retrotransposons; data not shown).

In addition, preliminary BLAST survey through GenBank using *Cg_HINE* elements indicated similarities in non-coding regions of some *C. gigas* genes. Regions of similarity correspond to entire elements or to their deletion derivatives (Supplementary Table S5). As an illustration, the *bmpr1* gene contains a whole *Cg_HINE* element with a short central array comprised of about two monomers similar to the CL2 consensus (80%). In the close vicinity of the *Gigasin-2* gene, a *Cg_HINE* element with 1.5 monomers similar to the CL6 consensus (73%) was found. The *bindin* gene incorporates three truncated *Cg_HINE* elements, one containing 8.7 monomers, averaging 76% similarity to the CL4 consensus sequence. The other two are ~ 4.5 kb distant and 99.7% identical one to the other. They contain arrays of 5.8 monomers, with 76% of average similarity to the CL1 consensus sequence.

Assessing the organizational patterns of *Cg_HINE* elements revealed their integration into assembled genomic sequences in both orientations. Because of the general association of examined TRs with *Cg_HINE* elements, element distribution was approximated by mapping sequences identical to arrays in CL1-13 onto *C. gigas* pseudochromosomes^45^. A dense interspersed pattern has been shown for each studied sequence, and no preference to any assembled chromosome or to any particular chromosomal segment could be detected (Supplementary Fig. S3).

## Discussion

In the present work we revealed sequences repeated in tandem in the genome assembly of the Pacific oyster *Crassostrea gigas*^23^. Because of our interest in understanding patterns and drivers of genome-wide dispersal of sequences that might be related to satDNAs, we limited our analysis to repeats between 100 and 500 bp in size, as most commonly found in satDNAs^46–48^, including bivalves’ satDNAs^30^. The search for TRs in genome assemblies introduces another limitation. Namely, long arrays of satDNAs, built of highly similar sequences repeated in tandem and characteristic for heterochromatic regions and centromeres^2^ are generally misrepresented or overlooked in genome outputs due to difficulties in discerning their sequential order and length^49^. However, such assemblies offer a reliable platform when a genome-wide “euchromatic” distribution of short arrays of TRs and sequences associated with them are specifically targeted^38,39,41,50,51^.

The 13 most abundant clusters of short arrays of satDNA-like TRs (CL1-13, Table 1) detected in our survey were assigned to the 0.85% of the assembled *C. gigas* genome, and form 18% of sequences repeated in tandem as anticipated by Zhang et al.^23^. Furthermore, clusters CL1-13 comprise 49% of all arrays we have detected as 100–500 bp long tandemly repeated monomers. The remaining arrays are either present in a small number per cluster, or are too different to be clustered at all, representing putative singletons. The 13 clusters analyzed in this study can therefore be considered as a representative sample in illustrating the genome-wide organizational patterns of satDNA-like TRs in the *C. gigas* genome assembly.

Monomer lengths in clusters CL1-13 exist in a narrow range, on average 140–180 bp. The arrays are mostly comprised of up to 5 monomers, and those with ≥ 5 make up only 9% of the studied sample, the longest array containing only 29 monomers. The model proposed by Scalvenzi and Pollet^52^ on *Xenopus* frogs predicts a predominance of short arrays of satDNA-like TRs in putative euchromatic genomic segments, as obtained in our analysis of the *C. gigas* genome assembly. According to this model, the limited array length is favored because of the inverse correlation between number of TRs and mobility of TEs that may be involved in their dispersal. This observation, however, does not exclude that some of the repeats can also be builders of long arrays of classical satDNAs, associated with heterochromatic fractions, not included in the genome assembly.

Analysis of flanking segments revealed regular association of short satDNA-like arrays in all 13 clusters with sequences that have structural signatures of *HINEs*, non-autonomous TEs of the *Helentron* superfamily. Accordingly, they also lack TSD and have oligo-T segments as the preferential insertion site^20,21,44^. The best-studied *HINE* elements are nevertheless *DINE-*1 and its derivatives, which are widespread in *Drosophila*^20,53,54^. Their centrally-located TRs can also be found in the form of classical satDNAs, hypothesizing the general role of TR-carrying *Helentrons* in satDNA expansion^39^.

The Cg170/HindIII satDNA family is the most abundant in *C. gigas*, comprising 1–4% of the genome, and located in the centromeric regions of some chromosomes^27–29^. It is therefore not surprising that monomer variants of this satDNA family appear in some of the *Cg_HINE* elements. The average number of repeats in the elements carrying Cg170/HindIII monomers is slightly higher than the number of repeats in other *Cg_HINE* elements, but with a clearly increased number of arrays that contain ≥ 5 monomers (Table 1). In this regard, we can speculate that non-Cg170/HindIII monomers enriched in arrays containing ≥ 5 monomers in *Cg_HINE_3* and *Cg_HINE_6* may represent repeats in expansion or copies of undetected classical satDNA candidate sequences in this species.

It must be noted that some elements classified as *HINE* were already detected in bivalves. The *Pearl* family was described in the cupped oyster *Crassostrea virginica* and the blood ark *Anadara trapezia* and it had been anticipated that related elements could also be present in *C. gigas*^32^. *Pearl* elements carry short arrays of up to six ~ 160 bp long central repeats, some of them being similar to monomers of classical satDNAs found in other bivalve species, including Cg170 of *C. gigas*. Of comparable architecture are also *DTC84* of the clam *Donax trunculus*^55^ and the element *MgE* in the Mediterranean mussel *Mytilus galloprovincialis*^56^.

Analysis of the relationships among 11 *Cg_HINE* elements in *C. gigas* can help to understand drivers of satDNA-like TR dispersal and evolution. In the studied sample, two characteristics turned out to be common to all of them. First, the orientation of an array with regard to the flanking sequences is always the same in every element, without exception, indicating a single event in TR formation. Second, association of satDNA-like sequences from a particular cluster with a specific pair of flanking sequences is consistent (but not vice-versa, see below). In addition, three groups of intraspecific relationships defined according to similarities among element modules (LF-array-RF) can be discerned.

Group 1 is formed by elements similar in flanking segments and in associated satDNA-like repeats (Fig. 4a). Accumulated mutations should allow subsequent spread of variants if they still retain the structural requirements needed for replication^16^. Concurrent accumulation of changes along the whole element length suggests a persistence of association between flanking modules and satDNA-like central repeats (in this case related to the Cg170/HindIII) emerging from the formation of the ancestral copy. It can be further hypothesized that changes accumulated in the array of satDNA-like TRs in the course of element evolution may be a source of the subfamilies of Cg170/HindIII satDNA. Comparably, *DINE-*1 elements in *Drosophila willistoni* diverged into three subtypes, with changes both in subTIRs and TRs^53^. Concurrent accumulation of differences along whole element lengths has also been observed in interspecies comparisons of *Pearl* elements *CvE* of *C. virginica* and *MgE* of the Mediterranean mussel *Mytilus galloprovincialis*^56^.

In group 2, related flanking segments incorporate unrelated satDNA-like TRs (Fig. 4b), indicating independent incorporation events into flanking sequences of common origin. At the interspecific level, TRs of different origin associated with related flanking segments were observed in *Drosophila DINE-*1 elements^53^.

Group 3 is formed by *Cg_HINE* elements unrelated in nucleotide sequences of all modules (Fig. 4c). Elements in this subset could therefore be considered as *HINE* families that arose independently in the genome. In addition, four *Pearl* elements detected in *C. virginica*^32^ are also of independent origin. It can be concluded that the existence of unrelated *Cg_HINE* elements indicate multiple, and probably not rare events of independent element acquisition.

A special case is represented by *C. gigas* elements that incorporate two arrays of TRs instead of only one, originating either from related or from unrelated clusters (Fig. 4a and c). Multiple central arrays can be formed by recombination of elements that share flanking modules but not the central repeats. An alternative hypothesis is that a junction fragment containing segments of two divergent satDNA arrays became a source of double arrays integrated into a single *Cg_HINE* element. Abrupt junctions between two repetitive sequences that may be candidates for such a scenario were observed in bivalves^31^, as well as in other species (for example^57–59^).

The genesis of TRs in TR-carrying elements can be explained in the light of two scenarios discussed by^52^. According to the first, precursor satDNA sequences are captured (“filled”) and further propagated by an element, while according to the second, TRs are formed from the element’s intrinsic sequences. Analysis of the acquisition of sequence segments by the insect *Helitrons* favor the filler DNA model, proposing that internal segments are integrated into an element by multiple insertions^17^. Such events might also explain the formation of double arrays as observed in the two *Cg_HINE* elements. In addition, divergent central repeats carried by related flanking modules can be a consequence of insertion of potential monomer segment(s) and concurrent excision of the previously existing sequence. This process can be based on motifs in satDNA sequences recognized by transposase-related proteins^60^, as explained for monomer replacements observed in a root-knot nematode satDNA^58^. Similar cut-and-replace events were also anticipated in our previous analysis of Cg170 satDNA junction regions^31^.

Autonomous *Helentrons* can be assumed to be putative partners of *Cg_HINE* elements. Nearly-perfect identity marked autonomous *Helentrons* as partners of three *DINE*-1 elements in *Drosophila*^20^. In this regard, a *Helentron*-type Rep motif 2, a signature of autonomous *Helentrons*, has been detected in *C. gigas* genome data^21^. We found 10 *C. gigas* autonomous elements that harbor the *Helentron*-type Rep motif 2 in Repbase but could not relate any of them with the *Cg_HINE*s (not shown), so the nature of their relationship, if any, remains unresolved. In addition, some *Cg_HINE*s were found integrated into repetitive regions that may represent other putative TEs or may be the result of segmental duplications. Frequent integration into other TEs as new drivers of spread is a feature expected for TR-carrying *Helentrons*^18^.

Analysis of the genomic dispersal of arrays in clusters CL1-13 revealed their apparently uniform distribution on all *C. gigas* pseudochromosomes, which comprise about 50% of the genome^45^. Our preliminary search indicated insertions of *Cg_HINE*s within non-coding regions of some genes. Functions of these genes are related to early embryogenesis (*bmpr1* gene)^61^, fertilization (*bindin* gene)^62^ and oyster defence system (*Ecsit* and *Gigasin-2* genes)^63^. It can be expected that also fragmented *Cg_HINE* elements, isolated arrays of TRs or monomer fragments could be found dispersed throughout the genome, affecting genes and/or their regulatory regions. *Helitrons* and *Helentrons* in general, whether they carry internal TRs or not, have a strong influence on gene expression, not only by frequent gene capturing but also by inserting themselves close to the gene^17,53,64^. Therefore, the abundance of satDNA-like TRs as parts of *Cg_HINE* elements suggests they have a high impact on *C. gigas* genome evolution and function.

## Conclusion

We searched the genome assembly of the Pacific oyster *C. gigas* for TRs that resemble satDNAs in their monomer length. In the euchromatic genome fraction the detected satDNA-like TRs are composed of only short arrays. The most abundant clusters of TRs (49% of all detected) have a monomer length in a narrow range of 140–180 bp, characteristic for classical satDNAs. We found the most abundant satDNA-like arrays of TRs in the *C. gigas* genome assembly integrated as central repeats of non-autonomous *HINE* elements. Each group of satDNA-like arrays is associated with element-specific flanking sequences, making altogether a unique *Cg_HINE* element. The ability to follow the evolution of whole elements indicates stability once a relationship between the satDNA-like TRs and their flanking sequences was established. Sequences related to the most abundant satDNA Cg170 of *C. gigas*^27,28^ were also found as short satDNA-like arrays in some of the 13 studied *Cg_HINE* elements, showing close interrelations between these two classes of repetitive sequences, TE and satDNAs. Information obtained in this study promote bivalves as a second model system, after *Drosophila*, in analysis of non-autonomous TR-carrying *Helentrons*, a still poorly understood group of TEs using RCR mechanism in their spread.

## Materials and methods

### Detection and grouping of tandem repeats

The assembled *C. gigas* genome sequence (oyster.v9.fa) was downloaded from https://gigadb.org/dataset/100030 and uploaded into Tandem Repeats Database (TRDB) available at https://tandem.bu.edu/cgibin/trdb/trdb.exe65. Tandem repeats (TRs) were extracted using default parameters: alignment parameters 2,7,7 (match, mismatch, indels) and 50 as the minimum alignment score. The resulting arrays of TRs were filtered using the following criteria: pattern size ≥ 100 and ≤ 500 bp and repeat copy number ≥ 2 (Supplementary Table 1). Filtered arrays were processed using the redundancy tool with redundancy by period set at 50% overlap to eliminate multiple reporting of repeats (i.e. in cases when one repeat is part of another one). The clustering tool, implemented in TRDB, was used to group arrays of TRs that share at least 70% similarity under the following conditions: cutoff value set to 70, heuristical algorithm, DUST (to filter low complexity regions), and PAM (default values) options included. Clusters were ordered in descending order according to the total number of arrays, so the first cluster, CL1, contained the highest number of arrays (Table 1). For further analysis, arrays belonging to a specific cluster were downloaded from TRDB, and processed in Geneious 9.0.4 (Biomatters, Ltd). Multiple sequence alignments were performed to obtain monomer consensus sequence for each cluster. *C. gigas* pseudochromosomes^45^ were annotated using local databases holding all arrays from clusters CL1-13. Only 100% identical arrays were annotated on the 10 pseudochromosomes.

### Defining DNA sequences flanking tandem repeats

In order to explore the genomic environment of recognized TRs, sequences surrounding arrays were extracted from the corresponding scaffolds using TRDB. Up to 4,000 bp was extracted from each array side, in dependence to the array position in the scaffold and the scaffold length. Arrays of TRs positioned at the very end of a scaffold were excluded from further analysis, as well as those containing stretches of “Ns” in the flanking sequence. The consensus left and right flanking sequences (LF and RF, respectively) have been determined in a series of multiple alignments, performed separately for arrays in each cluster. Using Map to Reference Tool implemented in Geneious 9.0.4 (Biomatters, Ltd), consensus LF and RF sequences were further used to anchor alignment of all LF-array-RF sequence segments in the corresponding cluster. In the following step, sequence segments matching consensus sequences were extracted and used in additional alignments, in order to obtain refined LF and RF consensus sequences for arrays of TRs in each cluster. In this way, number of flanking sequences used for the final derivation of LF and RF consensus sequences of each element was > 50%, and similarity according to the consensus was > 70% (Supplementary Table 2).

### Detection of substructures in sequences flanking tandem repeats

In order to detect substructures, LF and RF sequences of each array in the cluster were merged into chimeric constructs using a local script. Because of high sequence variability, instead of using consensus sequences, this was done for each LF and RF sequence pair, preserving their original orientation. Chimeric LF-RF sequence sets were imported into Inverted Repeats Database (IRDB; https://tandem.bu.edu/cgi-bin/irdb/irdb.exe). A search for inverted repeat (IR) was performed using default values, with the exception of alignment parameters set to 2,5,7 and the minimum alignment score set to 14. Results were filtered by position of IRs in a way that one of the pairs (left first index) is present in LF and the other (right first index) is in RF. Similarity of IRs was set to 90%. Sets of filtered IRs were downloaded and further analyzed by multiple sequence alignments. IRs that were the most prominent according to sequence similarity and abundance were selected, and, in order to check their appropriate positioning, annotated in chimeric LF-RF constructs using the Motif Search Tool implemented in Geneious 9.0.4 (Biomatters, Ltd), allowing 1 mismatch. This procedure enabled identification of IR and subterminal inverted repeat (subTIR) structures.

For palindrome search, we used programs *einverted* and *palindrome* in the EMBOSS package^66^ (https://www.hgmp.mrc.ac.uk/Software/EMBOSS/). Secondary structures formed by palindromes were predicted by the program *DNA fold* implemented in Geneious 9.0.4 (Biomatters, Ltd).

Tandem Repeat Finder v4.09.^67^ was used for the microsatellite detection and definition (alignment parameters set at 2,3,5). The microsatellite repeat consensus was determined by alignments of all microsatellite sequences within a cluster using MUSCLE (implemented in Geneious).

### Similarity search through databases

In order to determine similarities with known repetitive elements or other published sequences, monomer and flanking consensus sequences were queried through Repbase^68^ and NCBI GenBank Database. For local blast analysis, a collection of *C. gigas* repetitive elements was made by downloading sequences from Repbase.

### Empty site analysis

For each particular *Cg_HINE*, 10 to 20 elements with well-defined sequence segments were selected randomly and used in the empty site analysis. Sequences 50 bp upstream and downstream from the determined element ends were merged into chimeric constructs. These constructs were used as queries in a homology search through the genome assembly (Discontiguous Megablast, max E value = 10). At least 80% identity over 85% of query length was used as criterion for verification of a paralogous site.

### Exploring relationships among monomers in clusters

Arrays from clusters with highest mutual sequence similarity (CL1, CL2, CL10 and CL13), were randomly selected to obtain approximately 150 monomers from each of them. To avoid truncated monomers at array beginning and/or end, only those longer than 140 bp, and in the frame with the corresponding consensus sequence were selected. For the ~ 600 monomers, short FASTA headers were derived by renaming monomers in a way to distinguish the cluster and the array, as well as the monomer position in the array. MAFFT v7.017 type of alignment was used for further analyses^69^. The best-substitution model was identified by jModelTest 2.1.4.^70^. The best model was chosen according to the Akaike Information Criterion (TPM1uf + G). For the phylogenetic analysis PhyML 3.0.^71^ using 100 bootstrap replicates was run. The obtained maximum likelihood trees were displayed in Fig Tree 1.4.2. Available at https://tree.bio.ed.ac.uk/software/figtree/.

## Supplementary information

Supplementary information

Supplementary Table1

## Data Availability

All data generated or analyzed during this study are included in this published article and its supplementary information files.
