# Supplementary material for: Satellite DNA-like repeats are dispersed throughout the genome of the Pacific oyster Crassostrea gigas carried by Helentron non-autonomous mobile elements
Source: Sci Rep. 2020 Sep 15;10:15107. doi: 10.1038/s41598-020-71886-y (PMC7492417; doi:10.1038/s41598-020-71886-y)
Supplement: Supplementary file 1 — Supplementary information [file 41598_2020_71886_MOESM1_ESM.pdf]

Supplementary Information for:

Satellite DNA-like repeats are dispersed throughout the genome of the Pacific oyster *Crassostrea gigas* carried by *Helentron* non-autonomous mobile elements

Tanja Vojvoda Zeljko, Martina Pavlek, Nevenka Meštrović, and Miroslav Plohl*

Division of Molecular Biology, Ruđer Bošković Institute, Zagreb, Croatia

*Corresponding author:
Miroslav Plohl (plohl@irb.hr; Ruđer Bošković Institute, Bijenička 54, 10 000 Zagreb, Croatia; tel: +385 1 4564 083; fax: +385 1 4561 177)

This file includes Supplementary Figures S1 - S3 and Supplementary Tables S2 – S5.

SUPPLEMENTARY FIGURES

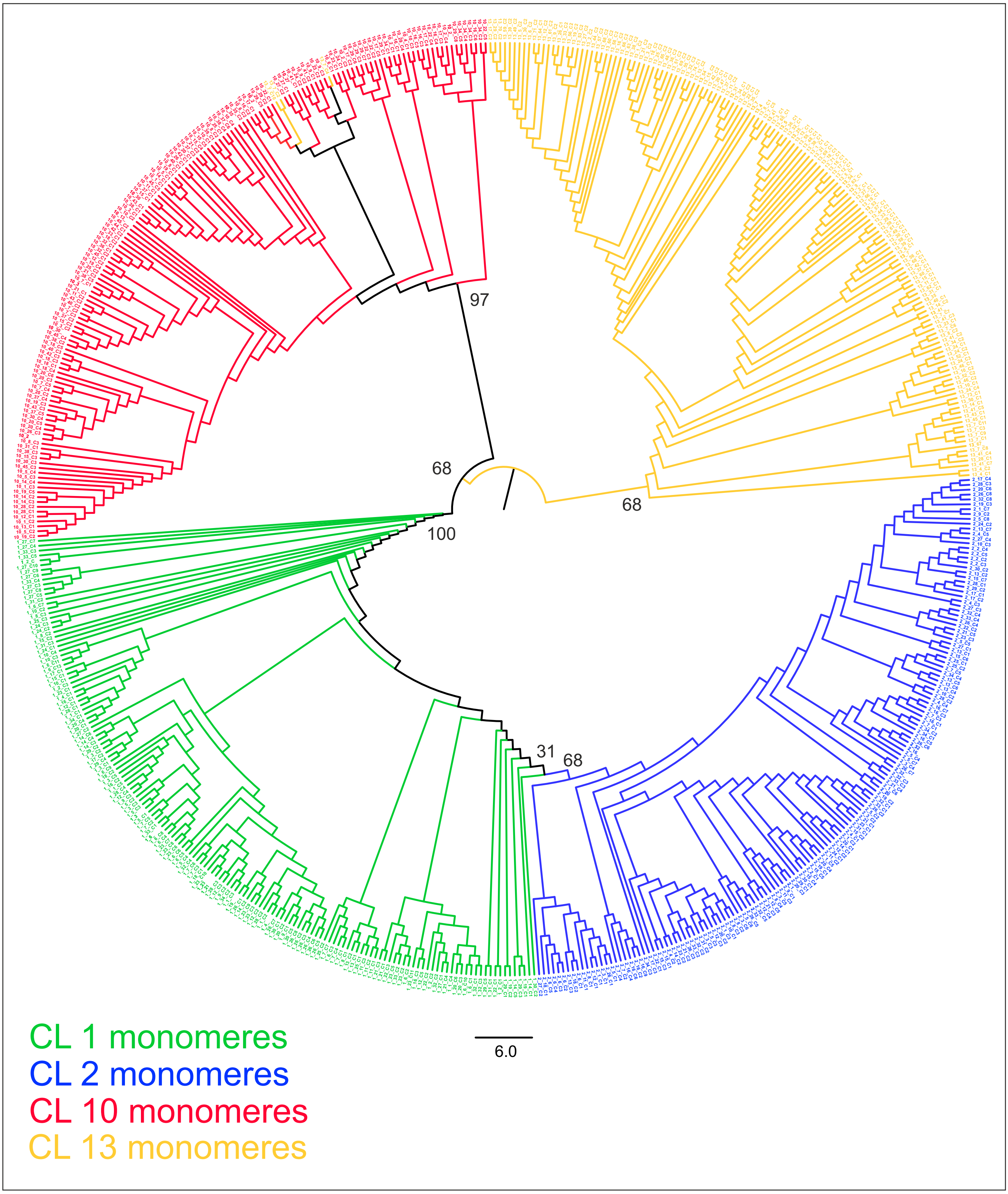

Supplementary Figure S1. Phylogenetic relationships of monomers in four clusters of related *Cg_HINE* central TRs. Shown is maximum-likelihood dendrogram based on nucleotide sequences belonging to monomers from clusters CL1, CL2, CL10 and CL13. Only branch support for the nodes separating main clades are shown.

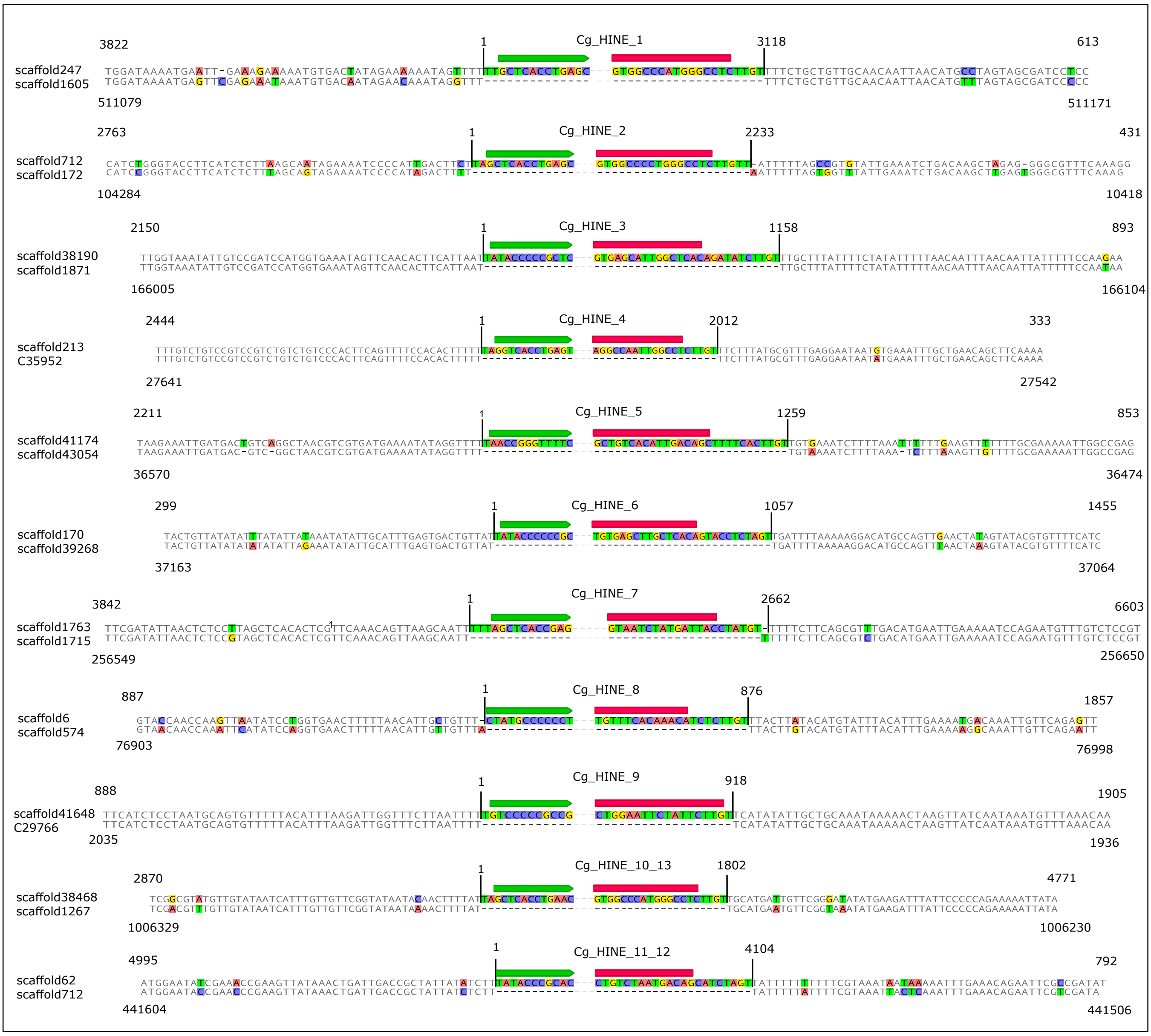

Supplementary Figure S2. Empty site analysis of 11 *Cg_HINE* elements. Chimeric constructs were made using 50 bp upstream and downstream from the determined *Cg_HINE* element ends and used as queries in a homology search through the *C. gigas* genome assembly. Only one representative result for each empty site is shown. In each alignment, upper sequence is query (with excluded *Cg_HINE* element insertion site presented in the figure), while lower is genomic scaffold with the relevant hit. Nucleotide positions are marked as in the genome assembly. For each query sequence, the exact *Cg_HINE* element position is presented, and its length is marked above the black lines marking the insertion sites. Only the 5' beginning of the element (including the subTIR part, green arrows) and the 3' end sequence (including the stem-loop structure, red lines) of a particular *Cg_HINE* element are shown.

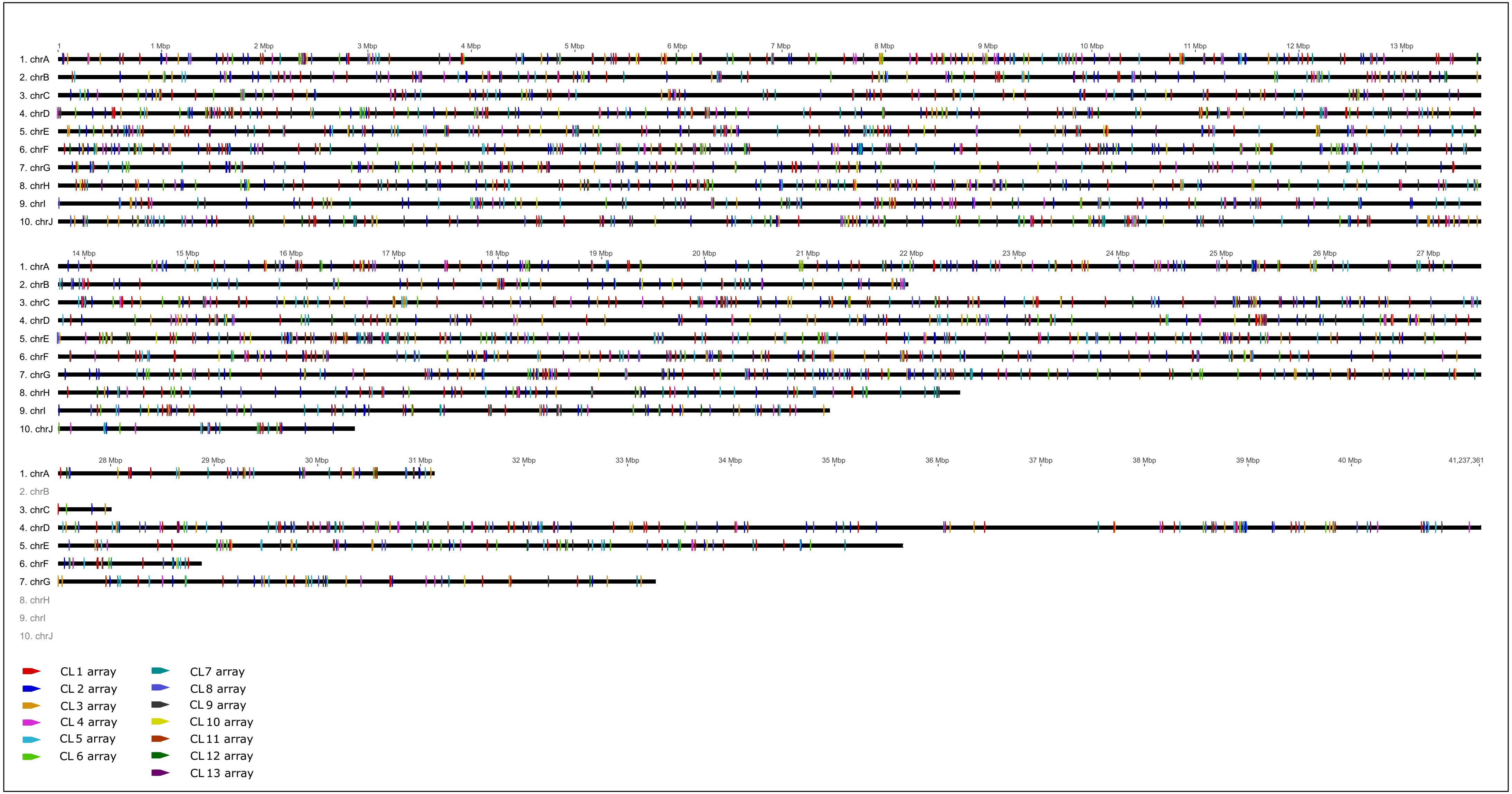

Supplementary Figure S3. Arrays from clusters CL1-13 mapped onto *C. gigas* pseudochromosomes. Arrangement of arrays is presented as an approximation of distribution of *Cg_HINE* elements. For this, a local database of CL1-13 arrays was used to map 100% identical sequences on the *C. gigas* pseudochromosomes[51].

SUPPLEMENTARY TABLES

Supplementary Table S2. Substructures of *Crassostrea gigas Cg_HINE* elements depicted in this work, and number and similarities of sequences used to derive LF and RF consensus segments. From the left flanking of specific Cg_HINE element, subterminal inverted repeats (subTIRs), inverted repeats (IRs) and microsatellite sequences are presented. SubTIR in the Cg_HINE right flanking (RF) is coupled with palindromic sequence which has the potential of forming stem – loop structure. Mismatches in substructures (subTIR, IR and palindromes) are shown in *italic*. For the microsatellite sequences, the minimal and the maximal number of the repeat unit is shown. S= G or C; Y= C or T; B= C, G or T; K= T or G. Central loop nucleotides in palindromic sequences are shown in brackets. The percentage of left flanking region (LF) and RF sequences was calculated as the total number of LF and RF sequences used in the alignments divided by the total number of arrays downloaded from TRDB (see also Table 1).

Cg_HINE element	SatDNA-like TRs in Cg_HINE	Subterminal inverted repeat (subTIR) in LF	Partial inverted repeat (IR) in LF	Microsatellite in LF	Palindromic sequences in RF (stem – loops)	The total number and the percentage of sequences used to derive Cg_HINE_LF consensus	The total number and the percentage of sequences used to derive Cg_HINE_RF consensus	Similarity of sequences in LF module (up to the microsatellite)	Similarity of sequences in RF module (up to 60 bp)	Number of LF-RF chimeric sequences in the search for substructures
Cg_HINE_1	CL1	GCTCAC*C*TGAGC	GCTCA*A*GTGAGC	$(GTCY)_{1-22}$	G*T*GGCCC(AT)GGGCC*T*C	820; 58.69%	916; 66%	83.8%	88.50%	1145
Cg_HINE_2	CL2			$(GTY)_{1-21}$	G*T*GGCCC(CT)GGGCC*T*C	772; 74.87%	691; 67%	83.5%	87.00%	934
Cg_HINE_3	CL3	ATACCCCC*G*CTC	GAG*A*GGGGGTAT	$(GTCY)_{2-26}$	GTGAGC(ATTG)GCTCAC	581; 82.64%	619; 88%	72.9%	90.80%	637
Cg_HINE_4	CL4	GGTCACCTGAGT	ACTCAGGTGACC	$(CGTCGTS)_{1-9}$	AGGCCA(AT)TGGCCT	460; 67.34%	493; 72%	77.4%	84.50%	643
Cg_HINE_5	CL5	AACCGG*G*TTTT*C*	*A*AAAA*T*CCGGTT	$(GGCG)_{1-10}$	GCTGTCA(CAT)TGACAG C	478; 74.33%	570; 89%	80.4%	87.20%	567
Cg_HINE_6	CL6	ATACCCCCC*GC*	*TG*GGGGGGTAT	$(GTCY)_{1-21}$	TGTGAGC(TT)GCTCACA	480; 80.4%	502; 84%	74.6%	86.90%	564
Cg_HINE_7	CL7	AGCTC*A*CCGAG	GG*G*GAGCT	$(GGCGTC)_{1-10}$	GTAATC(TGT)GATTAC	515; 91.3%	385; 68%	74.5%	83.50%	505
Cg_HINE_8	CL8	T*TATG*CC*C*CCCT	GG*A*GG*GCAT*A	$(GTCK)_{1-18}$	TGTTT(CAC)AAACA	418; 85.65%	425; 87%	80.4%	82.30%	470
Cg_HINE_9	CL9	GTCCCCCGCCG	GGGGAC*A*	$(GTCCGTGB)_{1-14}$	CA*G*GAAT(TCT)ATTC*T*TG	278; 90%	263; 85%	78.2%	87.80%	286
Cg_HINE_10_13	CL10	GCTCAC*C*TGAAC	GTTCA*A*GTGAGC	$(GTCY)_{1-25}$	GTGGCCCATGGGCCTC	307; 84%	322; 88%	88.5%	93.30%	324
	CL13							71.0%		
Cg_HINE_11_12	CL11	TATACCCG*C*AC	GT*T*CGGGTATA	$(GTCC)_{2-23}$	CTGTC(TAAT)GACAG	322; 87%	113; (31%)	85.4%	92.70%	307
	CL12									

Supplementary Table S3. Identity matrix of related satDNA-like repeats detected in this work and monomers of related "classical" satDNAs.

	Cg170_sat_Cons	HindIII_sat_Cons	CL1_ConsRep_rev	CL2_ConsRep_rev	CL10_ConsRep_rev	CL13_ConsRep_rev	CL4_ConsRep
**Cg170_sat_Cons**		94.28%	93.71%	92.17%	66.67%	65.77%	50.29%
**HindIII_sat_Cons**			92.51%	90.06%	69.94%	66.96%	52.65%
**CL1_ConsRep_rev**				89.82%	67.86%	65.48%	51.76%
**CL2_ConsRep_rev**					68.26%	66.07%	47.65%
**CL10_ConsRep_rev**						70.41%	47.95%
**CL13_ConsRep_rev**							45.88%
**CL4_ConsRep**							

Supplementary Table S4. Similarity of *Cg_HINE* modules with sequences deposited in Repbase. Consensus sequences of monomers („_Cons_Rep") and consensus sequences of left and right flanking regions („_Cons_LF"; „_Cons_RF") from clusters CL1-13 were separately queried through Repbase using CENSOR tool to align them against a reference collection of repeats. Base pairs which denote the exact beginning and end of a particular mobile element are shown in red. Similarity in the last column is calculated as Sim = match_count / (alignment_length - query_gap_length - subject_gap_length + gap_count).

Query name	From	To	Repbase library sequence name	From	To	Repeat class/sublass	Similarity between query and Repbase repeat fragment
CL1_Cons_LF	1	105	Helitron-N2_CGi	1	105	DNA/Helitron	0.9524
CL1_Cons_RF	1	60	Helitron-N2_CGi	2251	2310	DNA/Helitron	1.0000
CL1_Cons_Rep	1	167	Helitron-N55_CGi	1013	847	DNA/Helitron	1.0000
CL2_Cons_LF	1	88	Helitron-N2d_CGi	1	91	DNA/Helitron	0.9888
CL2_Cons_RF	1	100	Helitron-N2d_CGi	2320	2419	DNA/Helitron	1.0000
CL2_Cons_Rep	1	166	SAT-1_CGi	323	157	Simple/Sat/SAT	0.9940
CL3_Cons_LF	1	50	Helitron-N16_CGi	1	50	DNA/Helitron	1.0000
CL3_Cons_RF	1	58	Helitron-N16_CGi	1400	1457	DNA/Helitron	1.0000
CL3_Cons_Rep	2	167	Helitron-N25_CGi	443	608	DNA/Helitron	0.9940
CL4_Cons_LF	1	55	Helitron-N2e_CGi	1	55	DNA/Helitron	1.0000
CL4_Cons_RF	1	60	Helitron-N2e_CGi	1461	1521	DNA/Helitron	0.9672

CL4_Cons_Rep	3	170	Helitron-N2e_CGi	454	621	DNA/Helitron	0.9881
CL5_Cons_LF	1	66	Helitron-N45_CGi	1	70	DNA/Helitron	0.9851
CL5_Cons_RF	1	60	Helitron-N45_CGi	841	900	DNA/Helitron	0.9833
CL5_Cons_Rep	1	181	Helitron-N45_CGi	760	580	DNA/Helitron	0.9834
CL6_Cons_LF	1	77	Helitron-27_CGi	1	77	DNA/Helitron	0.9870
CL6_Cons_RF	1	60	Helitron-N18_CGi	970	1029	DNA/Helitron	1.0000
CL6_Cons_Rep	2	147	Helitron-N18_CGi	486	341	DNA/Helitron	0.9932
CL7_Cons_LF	1	50	DNA4-4B_CGi	1392	1441	DNA	0.9400
CL7_Cons_RF	1	60	DNA4-4_CGi	3641	3700	DNA	0.9667
CL7_Cons_Rep	2	178	Helitron-N12_CGi	572	748	DNA/Helitron	0.9435
CL8_Cons_LF	1	63	Helitron-N9_CGi	1	63	DNA/Helitron	1.0000
CL8_Cons_RF	1	49	Helitron-N9_CGi	897	946	DNA/Helitron	0.9800
CL8_Cons_Rep	2	162	Helitron-N9_CGi	395	555	DNA/Helitron	1.0000
CL9_Cons_LF	1	80	Helitron-N23_CGi	1	76	DNA/Helitron	0.9744
CL9_Cons_RF	1	56	Helitron-N23_CGi	884	939	DNA/Helitron	1.0000
CL9_Cons_Rep	2	173	Helitron-N23_CGi	689	518	DNA/Helitron	1.0000
CL10_Cons_LF	1	91	Helitron-N2C_CGi	1	91	DNA/Helitron	0.9890
CL10_Cons_RF	1	60	Helitron-N2C_CGi	2574	2633	DNA/Helitron	1.0000
CL10_Cons_Rep	2	167	Helitron-N2C_CGi	513	348	DNA/Helitron	1.0000
CL11_Cons_LF	1	82	Helitron-N17B_CGi	1	82	DNA/Helitron	0.9756
CL11_Cons_RF	1	60	Helitron-N17_CGi	4390	4449	DNA/Helitron	1.0000
CL11_Cons_Rep	1	162	Helitron-N17_CGi	618	781	DNA/Helitron	0.9755
CL12_Cons_LF	1	82	Helitron-N17B_CGi	1	82	DNA/Helitron	0.9756
CL12_Cons_RF	1	60	Helitron-N17_CGi	4390	4449	DNA/Helitron	1.0000
CL12_Cons_Rep	2	138	Helitron-N17_CGi	1180	1046	DNA/Helitron	0.9416
CL13_Cons_LF	1	93	Helitron-N2C_CGi	1	93	DNA/Helitron	0.9785
CL13_Cons_RF	1	60	Helitron-N2C_CGi	2574	2633	DNA/Helitron	1.0000
CL13_Cons_Rep	2	168	Helitron-N37_CGi	2616	2451	DNA/Helitron	0.9048

Supplementary Table S5.

Detected *Crassostrea gigas* genes containing *Cg_HINE* elements or their deletion derivatives. For each gene, coordinates where similarities were found are listed. For the left (LF) and right (RF) flanking regions similarities (%) to the consensus sequences of the *Cg_HINE* elements are given. The presence or absence of a specific *Cg_HINE* part is indicated with + / - signs.

Gene name (GenBank number)	Gene function	Location of *Cg_HINE* parts within gene	Left flanking region	5' subTIR	5' IR	5' microsatellite	Internal tandem repeats	Right flanking region	3' subTIR	3' stem-loop
Ecsit (gb|HQ225835.1)	Evolutionarily conserved signalling intermediate in *Toll* pathways	1662 - 3169. bp	-	-	-	+	7 repeats (93% similiar with CL1_ConsRep)	+ (98 % similar to CL1_Cons_RF)	+	+
Bindin (gb|EU307654.1)	In fertilization	2401 - 4126. bp	+ (66% similar to CL4_Cons_LF))	-	-	-	8.7 repeats (76% similar to CL4_ConsRep)	-	-	-
		12532 - 14723. bp	-	-	-	-	5.8 repeats (80% similar to CL1_ConsRep)	+ (90 % similar to CL1_Cons_RF)	+	+
		19257 - 21448. bp	-	-	-	-	5.8 repeats (81% similar to CL1_ConsRep)	+ (90 % similar to CL1_Cons_RF)	+	+
Bmpr 1 (emb|AJ577293.1)	In early embryonic development	6952 – 9174. bp	+ (87% similar to CL1_Cons_LF)	+	+	+	2 repeats (80% similar to CL1_ConsRep)	+ (77% similar to CL1_Cons_RF)	+	+
Gigasin-2 (emb|AJ582630.1)	Defensin-like antimicrobial peptide AMP-Gigasin 2	2180 - 2795. bp	+ (65% similar to CL6_Cons_LF)	+	+	+	1.5 repeats (76% similar to CL6_ConsRep)	+ (82% similar to CL6_Cons_RF)	+	+
